# Socioeconomic inequalities in coronary heart disease risk in older age: contribution of established and novel coronary risk factors

**DOI:** 10.1111/j.1538-7836.2009.03602.x

**Published:** 2009-11

**Authors:** S E RAMSAY, R W MORRIS, P H WHINCUP, O PAPACOSTA, A RUMLEY, L LENNON, G LOWE, S G WANNAMETHEE

**Affiliations:** * Division of Population HealthUCL, London; † Division of Community Health Sciences, St George’s University of LondonLondon; ‡ Division of Cardiovascular and Medical Sciences, University of GlasgowGlasgow, UK

**Keywords:** coronary heart disease, coronary risk factors, older age, social inequalities

## Abstract

*Background:*Evidence on socioeconomic inequalities in coronary heart disease (CHD) and their pathways in the elderly is limited. Little is also known about the contributions that novel coronary risk factors (particularly inflammatory/hemostatic markers) make to socioeconomic inequalities in CHD. *Objectives:*To examine the extent of socioeconomic inequalities in CHD in older age, and the contributions (relative and absolute) of established and novel coronary risk factors. *Methods:*A population-based cohort of 3761 British men aged 60–79 years was followed up for 6.5 years for CHD mortality and incidence (fatal and non-fatal). Social class was based on longest-held occupation recorded at 40–59 years. *Results:*There was a graded relationship between social class and CHD incidence. The hazard ratio for CHD incidence comparing social class V (unskilled workers) with social class I (professionals) was 2.70 [95% confidence interval (CI) 1.37–5.35; *P*-value for trend = 0.008]. This was reduced to 2.14 (95% CI 1.06–4.33; *P*-value for trend = 0.11) after adjustment for behavioral factors (cigarette smoking, physical activity, body mass index, and alcohol consumption), which explained 38% of the relative risk gradient (41% of absolute risk). Additional adjustment for inflammatory markers (C-reactive protein, interleukin-6, and von Willebrand factor) explained 55% of the relative risk gradient (59% of absolute risk). Blood pressure and lipids made little difference to these estimates; results were similar for CHD mortality. *Conclusions:*Socioeconomic inequalities in CHD persist in the elderly and are at least partly explained by behavioral risk factors; novel (inflammatory) coronary risk markers made some further contribution. Reducing inequalities in behavioral factors (especially cigarette smoking) could reduce these social inequalities by at least one-third.

## Introduction

Coronary heart disease (CHD) is an important cause of morbidity and mortality; the incidence and prevalence of CHD both rise steeply with increasing age [1]. Although there is a strong social class gradient in CHD risk in middle age [2–6], the extent of social inequalities in CHD in old age is not well established, and their implications for relative and absolute differences in CHD risk remain uncertain. Some reports suggest that the relative differences in overall and CHD mortality between socioeconomic groups may decrease with increasing age [3,6,7]. However, evidence specifically related to social inequalities in CHD in old age is limited.

The pathways through which social inequalities in CHD can operate in older age also remain uncertain. In middle age, established coronary risk factors, including smoking, physical inactivity, obesity, and hypertension, make appreciable, although limited, contributions to socioeconomic inequalities in CHD risk [8,9]. Novel risk factors, including inflammatory and hemostatic markers, are known to be associated with increased risk of coronary disease [10]. Some of these inflammatory markers, such as C-reactive protein (CRP), are also reported to be higher in lower socioeconomic groups, and therefore are increasingly hypothesized to be possible contributors to the association between socioeconomic position and CHD [11–13]. A study in middle-aged subjects has suggested that inflammatory markers such as fibrinogen can contribute to the relationship between socioeconomic position and CHD [14]. However, there is little information on these associations in later life.

A better understanding of the extent of social inequalities in CHD risk in later life (assessed both in relative and in absolute risks) and the role of underlying factors would inform appropriate initiatives and policy action to reduce health inequalities in older age. We therefore examined the extent of social inequalities in CHD incidence and mortality, using a prospective population-based study comprising older British men (aged 60–79 years). We also investigated the extent to which established behavioral [cigarette smoking, alcohol consumption, body mass index (BMI) and physical activity] and biological coronary risk factors (blood pressure and lipids) [15], and novel coronary risk factors [CRP, interleukin-6 (IL-6), and von Willebrand factor (VWF)] [10,16], contribute to socioeconomic differences in CHD in older men in both relative and absolute terms.

## Materials and methods

### Study design and population

The British Regional Heart Study is a prospective study of cardiovascular disease comprising a socially and geographically representative sample of men initially examined in 1978–1980 when aged 40–59 years, drawn from one general practice in each of 24 towns representing all major British regions [5]. In 1998–2000, the men, then aged 60–79 years, were invited to a 20-year reassessment, which included completion of a questionnaire, physical examination, and collection of a blood sample after a minimum 6-h fast; 4252 men (77% of surviving subjects) attended the examination, and 4094 men (74%) had at least one measurement of biological factors. For this report, follow-up data for CHD incidence and CHD mortality from 1998–2000 until 2006 was used. CHD incidence included non-fatal and fatal myocardial infarction (MI). Non-fatal MI was defined by the presence of at least two of the following characteristics, ascertained by regular 2-yearly reviews of general practitioner records – severe prolonged chest pain, electrocardiographic evidence of MI, and cardiac enzyme changes consistent with MI. Data on mortality were obtained throughout the follow-up from the National Health Service Central Register. Fatal MIs were identified as deaths with *International Classification of Diseases*, 9th revision (ICD-9) codes of 410–414 (equivalent to ICD-10 codes I20–I25).

### Social class

The longest-held occupation of subjects at study entry (aged 40–59 years) was used to define social class using the Registrar General’s Social Class Classification – I (professionals, e.g. physicians and engineers); II (managerial, e.g. teachers and sales managers); III non-manual (semiskilled non-manual, e.g. clerks and shop assistants); III manual (semiskilled manual, e.g. bricklayers); IV (partly skilled, e.g. postmen); and V (unskilled, e.g. porters and general laborers). Information on social class was not available for eight subjects. Men in the armed forces were excluded from analyses [112 (2.6%)]. Therefore, information on social class in these analyses was restricted to 4132 men.

### Behavioral and biological risk factors

Through the combination of information collected in 1998–2000 and previous questionnaires, subjects were classified as never smokers, long-term ex-smokers (> 20 years), ex-smokers who stopped smoking 15–20 years ago, ex-smokers who stopped smoking 10–15 years ago, ex-smokers who stopped smoking 5–10 years ago, ex-smokers who stopped smoking within 5 years, and current smokers [17]. On the basis of their alcohol intake in 1998–2000, subjects were classified as follows: none, occasional (< 1 drink per week), light (1–15 drinks per week), moderate (16–42 drinks per week), and heavy (> 42 drinks per week – daily or most days of the week) [17]. One drink was defined as half a pint of beer, a glass of wine, or a tot of spirit (8–10 g). Physical activity scores based on frequency and type of activity were as follows: none, occasional, light, moderate, moderately vigorous, and vigorous [18]. None/occasional activity was classified as ‘inactive’. BMI was calculated as weight per height squared (kg/m^2^). Measurements of blood pressure, triglycerides, high-density lipoprotein cholesterol (HDL-C) and low-density lipoprotein cholesterol (LDL-C) have been described previously [17].

### Novel coronary risk factors

Details of CRP, IL-6 and VWF have been described previously [19]. CRP and IL-6 were chosen because these inflammatory markers have been reported to be novel coronary risk factors [16,20], and are increasingly being hypothesized to be possible explanations for socioeconomic inequalities in CHD [13,21,22]. The association of VWF with social class has been previously observed to be independent of established coronary risk factors in our subjects [19].

### Statistical analyses

Triglyceride, CRP and IL-6 distributions were positively skewed and required log transformation. Cox proportional hazards models were used to calculate hazard ratios with 95% confidence intervals (CIs) for CHD incidence and mortality according to social class. Social class I was the reference category. Social class was also fitted as a continuous variable in the Cox models to obtain regression coefficients and hazard ratios (95% CI) per unit increase in social class and the *P*-value for trend associated with this. The proportionality assumption for the Cox models was assessed by testing the Schoenfeld residuals [23], and was found to be valid. Cox models included age and behavioral risk factors, and were further adjusted for biological risk factors. Novel risk factors (CRP, IL-6, and VWF) were individually added into the model to assess their contribution in addition to behavioral factors. For the adjustments, age, BMI, systolic blood pressure, triglycerides, LDL-C, HDL-C, CRP, IL-6 and VWF were fitted as continuous variables; social class (six levels), smoking (six levels), physical activity (five levels) and alcohol intake (five levels) were fitted as ordinal variables.

The contribution of risk factors to the relative social class difference was calculated with the formula [(*β*_0_ − *β*_1_)/*β*_0_] × 100, where *β*_0_ was age-adjusted log hazard ratio per unit increase in social class, and *β*_1_ was log hazard ratio adjusted for different risk factors. Survival probability at 6.5 years, the mean survival time, was calculated for each social class by applying average levels of age and risk factors to all social classes in each of the above models. Event probability for CHD incidence and mortality was calculated as 1 – survival probability, expressed as a percentage. Absolute social class difference explained by risk factors was calculated with the formula [(AD_0_ − AD_1_)/AD_0_] × 100, where AD_0_ was the age-adjusted absolute difference in event probability between social classes I and V, and AD_1_ was the difference in event probability between social classes I and V adjusted for different risk factors. Approximate 95% CIs for the estimates of relative and absolute risk explained in each model were calculated using bias-corrected bootstrap resampling of size 1000 to estimate the upper and lower limits [24].

Population attributable risk fraction (PARF) comparing manual with non-manual social class was calculated for CHD incidence and CHD mortality, using the formula *p*(RR − 1)/[1 + *p*(RR − 1)], where *p* was the proportion of manual social class in the study population, and RR was the relative risk for CHD for manual as compared with non-manual social classes (hazard ratios from Cox regression models were used for the relative risks). PARF adjusted for coronary risk factors was obtained using hazard ratios adjusted for the different risk factors. All analyses were carried out using sas (version 9.1 (version 9.1, SAS Institution Inc., Cary, NC, USA)) and stata (version 10.1, StataCorp, College Station, TX, USA).

## Results

Among 4132 men aged 60–79 years who attended the re-examination, complete information on all coronary risk factors was available for 3761. The age and social class distribution of this group did not differ from that of 371 men with missing data; both groups had a mean age of 69 years and contained 48% of subjects of non-manual social classes. The proportion of smokers was slightly greater (15%) in the group with missing data than in the group without missing data (12%); mean BMI and systolic blood pressure were similar in the two groups. Missing information was largely due to unavailability of blood measurements in men who declined to provide blood samples.

Among 3761 men, 274 incident (non-fatal and fatal) CHD cases had occurred over a mean 6.5 years of follow-up, of which 191 were CHD deaths. Table 1 shows the distribution of coronary risk factors across social class groups. Greater percentages of current smokers and physically inactive and obese men, but a lower percentage of never smokers, were observed in manual than in non-manual social classes. Men of manual social classes had higher mean levels of triglycerides, CRP, IL-6 and VWF and lower levels of HDL-C than non-manual groups.

**Table 1:** Social class distribution of behavioral and biological coronary risk factors and inflammatory markers in British men aged 60–79 years in 1998–2000

	Social class I (*n* = 372)	Social class II (*n* = 1035)	Social class III non-manual (*n* = 381)	Social class III manual (*n* = 1525)	Social class IV (*n* = 336)	Social class V (*n* = 112)
Current smokers, *n* (%)	23 (6)	90 (9)	35 (9)	234 (15)	72 (21)	13 (12)
Never smokers, *n* (%)	181 (49)	349 (34)	131 (34)	341 (22)	86 (26)	24 (21)
Heavy/moderate drinkers, *n* (%)	91 (25)	206 (20)	55 (15)	267 (18)	57 (17)	25 (23)
Physically inactive, *n* (%)	92 (25)	298 (30)	138 (37)	520 (36)	131 (40)	51 (47)
Mean BMI [kg m^−2^ (SD)]	26.2 (3.39)	26.7 (3.52)	26.8 (3.53)	27.3 (3.81)	27.1 (4.09)	27.6 (4.19)
Obese (BMI > 30 kg m^−2^), *n* (%)	40 (11)	140 (14)	56 (15)	288 (19)	64 (19)	28 (25)
Mean systolic blood pressure [mmHg (SD)]	148 (25)	149 (25)	150 (23)	149 (24)	150 (25)	149 (24)
Geometric mean triglycerides [mmol L^−1^ (95% range)]	1.51 (0.63–3.65)	1.55 (0.65–3.66)	1.62 (0.67–3.91)	1.66 (0.66–4.17)	1.57 (0.6–4.09)	1.58 (0.54–4.63)
Mean HDL cholesterol [mmol L^−1^ (SD)]	1.40 (0.37)	1.34 (0.33)	1.31 (0.33)	1.30 (0.34)	1.33 (0.34)	1.30 (0.38)
Mean LDL cholesterol [mmol L^−1^ (SD)]	3.93 (0.97)	3.91 (0.99)	3.93 (0.98)	3.88 (0.97)	3.86 (0.97)	3.89 (0.83)
Mean cholesterol [mmol L^−1^ (SD)]	6.06 (1.07)	5.99 (1.08)	6.04 (1.10)	5.99 (1.07)	5.94 (1.09)	6.01 (1.15)
Geometric mean CRP [mg L^−1^ (95% range)]	1.23 (0.16–9.25)	1.50 (0.18–12.44)	1.86 (0.22–16.06)	1.97 (0.22–17.66)	2.17 (0.23–20.29)	2.00 (0.21–18.73)
Mean VWF [IU dL^−1^ (SD)]	132 (45)	135 (45)	141 (45)	142 (46)	151 (47)	153 (53)
Geometric mean IL-6 [pg mL^−1^ (95% range)]	1.97 (0.60–6.52)	2.23 (0.63–7.80)	2.48 (0.69–8.88)	2.69 (0.71–10.20)	2.75 (0.78–9.63)	2.80 (0.68–11.49)

BMI, body mass index; CRP, C-reactive protein; HDL, high-density lipoprotein; IL-6, interleukin-6; LDL, low-density lipoprotein; SD, standard deviation; VWF, von Willebrand factor.

Table 2 shows hazard ratios (with 95% CIs) for CHD incidence and mortality according to social class and the effect of adjustment for risk factors. A social class gradient in the risk of CHD incidence and mortality was observed, with the hazard ratio increasing from social class I (professionals) to social class V (age-adjusted *P*-value for trend was 0.008 for CHD incidence and 0.02 for CHD mortality). In age-adjusted analyses, social class V (unskilled workers) had more than two and a half times increased incidence and mortality from CHD as compared with social class I. Age-adjusted CHD risk (incidence and mortality) increased by about 1.14 for every unit increase in social class (Table 2). Adjusting for behavioral risk factors attenuated this increased risk of CHD incidence and mortality; most of this attenuation (20%) was caused by cigarette smoking. Behavioral risk factors explained 38% (95% bootstrap CI 12–166%) of the increased hazard ratio for CHD incidence and 39% (95% CI 8–236%) of that for CHD mortality in lower social class groups. Further adjustment for biological risk factors did not alter these results materially. Adjustment, individually for CRP, IL-6 or VWF in addition to behavioral risk factors, further attenuated the effect of social class – CRP contributed to 46%, and IL-6 and VWF contributed to 47%, of the relative difference in CHD incidence between social class groups. All of the behavioral, biological and novel coronary risk factors together explained 55% (95% CI 22–214%) of the increased hazard ratio for CHD incidence, and 56% (95% CI 15–273%) of the hazard ratio for CHD mortality in lower social classes.

**Table 2:** Hazard ratio and 95% confidence interval for coronary heart disease (CHD) incidence and mortality and the contribution of established and novel coronary risk factors to relative social class inequalities in men aged 60–79 years followed up from 1998 to 2000

Social class	Age-adjusted	Age and behavioral factors*	Age, and behavioral and biological risk factors†	Age, behavioral factors, and CRP	Age, behavioral factors, and IL-6	Age, behavioral factors, and VWF	All risk factors
CHD incidence							
I	1.00	1.00	1.00	1.00	1.00	1.00	1.00
II	1.21 (0.74–1.99)	1.13 (0.68–1.86)	1.10 (0.67–1.82)	1.09 (0.66–1.80)	1.12 (0.68–1.85)	1.12 (0.68–1.85)	1.08 (0.65–1.78)
IIINM	1.26 (0.71–2.25)	1.16 (0.65–2.08)	1.12 (0.62–2.01)	1.09 (0.61–1.96)	1.13 (0.63–2.02)	1.13 (0.63–2.02)	1.05 (0.58–1.88)
IIIM	1.40 (0.87–2.26)	1.23 (0.75–2.00)	1.20 (0.74–1.95)	1.16 (0.71–1.89)	1.16 (0.71–1.89)	1.19 (0.73–1.95)	1.11 (0.68–1.82)
IV	1.60 (0.90–2.84)	1.22 (0.68–2.21)	1.23 (0.68–2.22)	1.17 (0.65–2.11)	1.19 (0.66–2.15)	1.17 (0.65–2.11)	1.14 (0.63–2.06)
V	2.70 (1.37–5.35)	2.14 (1.06–4.33)	2.08 (1.03–4.19)	2.04 (1.01–4.11)	2.08 (1.03–4.18)	2.02 (0.97–4.08)	1.88 (0.93–3.81)
Per unit social class	1.14 (1.04–1.25)	1.08 (0.98–1.19)	1.08 (0.98–1.19)	1.07 (0.97–1.18)	1.07 (0.97–1.18)	1.07 (0.97–1.18)	1.06 (0.96–1.17)
*P*-value for trend‡	0.008	0.11	0.11	0.16	0.17	0.17	0.25
Percentage attenuation in age-adjustedrelative risk per unit social class afteradjustment for risk factors§		38	38	46	47	47	55

CHD mortality							

I	1.00	1.00	1.00	1.00	1.00	1.00	
II	1.20 (0.66–2.17)	1.11 (0.61–2.01)	1.09 (0.60–1.98)	1.06 (0.58–1.93)	1.11 (0.61–2.03)	1.07 (0.60–1.98)	1.07 (0.59–1.95)
IIINM	0.96 (0.47–1.99)	0.86 (0.41–1.79)	0.83 (0.40–1.74)	0.78 (0.37–1.64)	0.83 (0.40–1.73)	0.81 (0.39–1.70)	0.77 (0.37–1.61)
IIIM	1.39 (0.79–2.46)	1.19 (0.66–2.13)	1.18 (0.66–2.11)	1.11 (0.62–1.99)	1.12 (0.62–2.01)	1.14 (0.64–2.05)	1.07 (0.60–1.92)
IV	1.59 (0.80–3.15)	1.21 (0.60–2.44)	1.23 (0.61–2.48)	1.15 (0.57–2.32)	1.20 (0.60–2.43)	1.14 (0.56–2.30)	1.14 (0.56–2.31)
V	2.77 (1.23–6.24)	2.13 (0.92–4.91)	2.06 (0.90–4.76)	2.01 (0.87–4.65)	2.12 (0.92–4.88)	2.00 (0.87–4.63)	1.88 (0.81–4.36)
Per unit social class	1.15 (1.02–1.28)	1.09 (0.97–1.22)	1.09 (0.97–1.23)	1.08 (0.96–1.21)	1.07 (0.95–1.21)	1.07 (0.95–1.21)	1.06 (0.94–1.20)
*P*-value for trend‡	0.02	0.17	0.16	0.23	0.24	0.24	0.32
Percentage attenuation in age-adjusted relative risk per unit social class after adjustment for risk factors§		39%	37%	46%	46%	48%	56%

CRP, C-reactive protein; IL-6, interleukin-6; VWF, von Willebrand factor.

* Behavioral factors included smoking, alcohol consumption, physical activity, and body mass index.

† Biological risk factors included systolic blood pressure, triglycerides, low-density lipoprotein cholesterol, and high-density lipoprotein cholesterol.

‡ *P*-value for trend obtained from Cox regression model with social class fitted as a continuous variable.

§ [(*β*_0_ − *β*_1_)/*β*_0_] × 100; *β*_0_ is age-adjusted log hazard ratio per unit increase in social class, and *β*_1_ is log hazard ratio per unit increase in social class additionally adjusted for risk factors.

The event probability for CHD incidence and CHD mortality at 6.5 years was graded according to social class (Table 3); social class I had the lowest event probability, and social class V had the highest. Adjustment for behavioral risk factors explained 41% (95% CI 18–132%) of the absolute risk difference between social classes. Further adjustment for biological risk factors did not substantially add to the contribution of behavioral factors. In addition to behavioral risk factors, adjustment for CRP explained 49% of the absolute social class difference, and IL-6 and VWF explained 51% each. All of these risk factors together contributed 59% (95% CI 33–312%) of the absolute social class difference in risk of CHD incidence, and 63% (95% CI − 153–162%) of that for CHD mortality.

**Table 3:** Event probability at 6.5 years for coronary heart disease (CHD) incidence and mortality and the contribution of established and novel coronary risk factors to the absolute social class difference in event probability

Social class	Age-adjusted	Age and behavioral factors*	Age, and behavioral and biological risk factors†	Age, behavioral factors, and CRP	Age, behavioral factors, and IL-6	Age, behavioral factors, and VWF	All risk factors
CHD incidence							
I	4.74	5.08	4.92	5.12	5.12	5.16	5.05
II	5.38	5.50	5.33	5.49	5.48	5.52	5.35
IIINM	6.11	5.95	5.78	5.88	5.86	5.91	5.67
IIIM	6.93	6.44	6.26	6.30	6.71	6.32	6.01
IV	7.86	6.96	6.78	6.75	6.55	6.75	6.37
V	8.90	7.53	7.34	7.23	7.17	7.22	6.75
Percentage attenuation inabsolute difference betweensocial classes I and V afteradjustment for risk factors‡		41%	42%	49%	51%	51%	59%

CHD mortality							

I	2.85	2.91	2.81	2.91	2.87	2.95	2.83
II	3.27	3.17	3.07	3.13	3.08	3.17	3.01
IIINM	3.75	3.45	3.34	3.37	3.32	3.40	3.20
IIIM	4.30	3.75	3.65	3.64	3.56	3.66	3.40
IV	4.92	4.07	3.98	3.92	3.83	3.93	3.62
V	5.63	4.43	4.34	4.22	4.12	4.22	3.85
Percentage attenuation in absolute difference between social classes I and V after adjustment for risk factors‡		45%	45%	53%	55%	55%	63%

CRP, C-reactive protein; IL-6, interleukin-6; VWF, von Willebrand factor.

* Behavioral factors included smoking, alcohol consumption, physical activity, and body mass index.

† Biological risk factors included systolic blood pressure, triglycerides, low-density lipoprotein cholesterol, and high-density lipoprotein cholesterol.

‡ (AD_0_ − AD_1_)/AD_0_ × 100; AD_0_ is age-adjusted absolute difference in event probability between social classes I and V; AD1 is absolute difference in event probability adjusted for risk factors.

Table 4 shows PARFs from manual social classes for CHD incidence and CHD mortality; these indicate the population risk for CHD incidence or mortality attributable to the excess risk in manual as compared with non-manual social classes. Table 4 also shows the PARF for CHD adjusted for different risk factors and the contribution of these risk factors to reducing the PARF from manual social class. The PARFs for manual vs. non-manual social classes were 12% for CHD incidence and 15% for CHD mortality. Adjustment for behavioral risk factors reduced the PARFs to 7% for CHD incidence and 10% for CHD mortality, thus accounting for 41% of the PARF (manual vs. non-manual groups) for CHD incidence and 34% of that for CHD mortality. Further adjustment for biological risk factors did not alter these attributable risk fractions. Adjusting for CRP, IL-6 and VWF individually in addition to behavioral factors further reduced the PARF slightly; all together, these risk factors with behavioral factors explained 56% of the reduction in PARF from manual social class for CHD incidence and 52% of that for CHD mortality.

**Table 4:** Population attributable risk fraction (PARF) from socioeconomic differences between manual and non-manual social class for coronary heart disease (CHD) incidence and mortality

	PARF						
	Age-adjusted	Age and behavioral factors*	Age, and behavioral and biological risk factors†	Age, behavioral factors, and CRP	Age, behavioral factors, and IL-6	Age, behavioral factors, and VWF	All risk factors
PARF (%) – CHD incidence	12	7	7	6	5	6	5
% PARF explained by risk factors‡		41	41	52	56	52	56
PARF (%) – CHD mortality	15	10	10	9	7	9	7
% PARF explained by risk factors‡		34	34	43	52	43	52

CRP, C-reactive protein; IL-6, interleukin-6; VWF, von Willebrand factor.

* Behavioral factors included smoking, alcohol consumption, physical activity, and body mass index.

† Biological risk factors included systolic blood pressure, triglycerides, low-density lipoprotein cholesterol, and high-density lipoprotein cholesterol.

‡ (Unadjusted PARF − adjusted PARF)/unadjusted PARF × 100.

## Discussion

In this prospective study of men aged 60–79 years, marked socioeconomic inequalities in CHD were present in older age; a nearly three-fold greater risk of CHD was present in the lowest than in the highest social class, and the absolute difference was 4%. Appreciable proportions of both increased relative and absolute risks were explained by behavioral factors, especially cigarette smoking, and also BMI, physical activity, and alcohol consumption. Novel coronary risk factors, including CRP, IL-6 and VWF, also accounted for some of the CHD inequalities in older age.

To our knowledge, this is the first study reporting relative and absolute contributions of established as well as novel risk factors to social inequalities in CHD risk in older subjects (60–79 years) with a mean age over 65 years. This study was carried out in a socioeconomically representative cohort of older British men with a high completeness of follow-up (98%; loss to follow-up was mostly due to emigration from the country). Missing data for a small proportion of subjects (*n* = 371; 9%) may have resulted in bias due to selection of healthier subjects, although this is unlikely, as the main reason for missing data was subjects declining to provide blood samples. Moreover, the distribution of social class and other characteristics, including age, BMI, and systolic blood pressure, was similar in subjects with and without missing data. The social class measure used, based on longest-held occupation during middle age (40–59 years), is a particularly stable indicator of socioeconomic position during adult life through to old age; a repeat assessment of social class before retirement indicated a very low proportion (8%) of marked social class change [25]. The use of such a measure overcomes the difficulties of measuring social class directly in later life [26]. However, the study population comprised only men, mostly White Caucasian, thus limiting the generalizability of findings to women and other ethnic groups. Given the dynamic nature of the association between socioeconomic position and coronary risk, which differs across time and place [27–29], caution needs to be exercised in applying the findings of this study, particularly in countries with economies in transition. Nevertheless, our findings are consistent with other studies showing socioeconomic differences in coronary risk and risk factors in other ethnic groups [6,30,31] and older women [32–34]. Although limited numbers of events resulted in wide bootstrap CIs, it is nevertheless useful to have estimates to quantify the likely contribution of coronary risk factors to socioeconomic inequalities in CHD.

The presence of social inequalities in CHD in older age in our study is consistent with previous studies, which reported an approximately 50% increase in relative risk of CHD in lower as compared with higher socioeconomic groups [7,33,35]. Previous studies in older populations have not reported the magnitude of socioeconomic differences in CHD in absolute terms. In the present study, the absolute difference in CHD risk between the highest and lowest social classes was 4%; for every 100 men followed up for a mean period of 6.5 years in each of the highest and lowest social classes, four extra CHD events were expected in the lowest social class group.

Social class differences in behavioral risk factors, including cigarette smoking (the most important single factor), physical inactivity, BMI, and alcohol consumption, made an important contribution to explaining the increased relative (38%) and absolute (over 40%) risk of CHD in lower social classes. In an older Swedish population, adjustment for coronary risk factors (smoking, physical activity, BMI, hypertension, and diabetes) attenuated this increased risk [33], whereas in a study comprising older Danish men (mean age 63 years), adjustment for established cardiovascular risk factors (smoking, blood pressure, lipids, and physical activity) made only a small contribution to the relative social difference in CHD risk [35] – inconsistencies between these studies in the effect of coronary risk factors may be due to weaker social class differences in cigarette smoking in the Danish study [35]. In the present study, biological coronary risk factors, such as blood pressure, HDL-C, LDL-C, and triglycerides, made little contribution, reflecting their weak social class distribution; their potential to reduce overall levels of coronary risk in older age, however, is still important [36,37]. Novel cardiovascular risk factors (CRP, IL-6, and VWF) explained an additional 10% of the relative social inequalities in CHD risk. The contribution of these inflammatory and hemostatic markers may reflect increased morbidity and accumulation of adverse coronary risk factors such as smoking, physical inactivity, dyslipidemia, and hypertension, associated with ageing [17,18,38,39]. Taken together, both health behaviors and novel risk factors together explained about 55% of relative and about 60% of absolute social class inequalities in CHD. Previous studies in older populations have not investigated the possible contribution of novel coronary risk factors, such as inflammatory markers, to socioeconomic inequalities in CHD. The Women’s Health Study showed that, in middle-aged women, CRP and fibrinogen explained little of the socioeconomic differences in cardiovascular disease in addition to the effect of traditional coronary risk factors [11]. In the Scottish Heart Health Study, fibrinogen did not influence social differences in CHD in middle-aged women, although it played a more important role in men [14]. The role of other possible mechanisms, such as oxidative stress, which has recently been hypothesized to be a possible link between socioeconomic position and coronary risk [40], was not investigated in the present study and needs further exploration.

Results PARFs showed that behavioral risk factors also made the largest contribution to reducing the population risk for CHD attributable to manual social classes, and novel coronary risk factors made some additional contribution. If manual social classes had the same CHD risk as non-manual groups, 12% of all CHD events could have been prevented. This population risk attributable to social class differences would be reduced to 7% if behavioral factors in manual social classes were similar to those in non-manual groups – implying a 41% contribution of behavioral risk factors to the population risk for CHD attributable to manual social classes.

## Implications and conclusions

Socioeconomic inequalities in CHD risk are present at older ages. Emerging coronary risk factors, to an extent, but predominantly behavioral factors (particularly cigarette smoking) are important determinants of social inequalities in CHD in the elderly. The substantial contribution of emerging and behavioral risk factors together to the absolute risk difference between social classes in our results indicates their potentially important public health impact on reducing CHD inequalities in older people. Social inequalities in CHD in older age could be narrowed by at least one-third through reductions in levels of behavioral risk factors including cigarette smoking, BMI, and physical inactivity – the potential of behavioral risk factors is likely to be even greater given the likelihood of measurement errors and failure to capture the role of risk factors across the life course. These factors are also important because of their strong influence on novel coronary risk factors such as inflammatory markers [17,18,39], which additionally contributed to the social inequalities in CHD. The wider social, cultural, political and material societal context, along with disadvantaged socioeconomic conditions across the life course, is known to be important in the origin of adverse health behaviors [41]. Policy efforts in improving levels of behavioral coronary risk factors can significantly reduce the extent of social inequalities in heart disease in older populations.
